# Analysis of Cohesion and Collective Efficacy Profiles for the Performance of Soccer Players

**DOI:** 10.2478/hukin-2013-0085

**Published:** 2013-12-31

**Authors:** Francisco M. Leo, Pedro A. Sánchez-Miguel, David Sánchez-Oliva, Diana Amado, Tomás García-Calvo

**Affiliations:** 1 Faculty of Sport Science, University of Extremadura.; 2 Faculty of Education, University of Extremadura.

**Keywords:** Profiles, cohesion, efficacy, performance, soccer

## Abstract

The principal aims of the study were to define different profiles of cohesion and perceived efficacy in soccer players and to measure their differences in performance. The subjects were 235 soccer players in the under-18 category who played in the National League in Spain and 15 coaches whose ages ranged from 29 to 45 years. Diverse instruments to assess cohesion, perceived efficacy, and expectations of success were used in the study. Moreover, we measured playing time and performance. The results of the study proved the existence of four cohesion and efficacy profiles that presented significant differences in expectations of success, playing time, and performance. Furthermore, significant differences were found in the distribution of players in the teams as a function of performance. The main conclusion of this study is that soccer players with higher cohesion and collective efficacy levels belonged to teams that completed the season at the top-level classification. In contrast, athletes with low cohesion and collective efficacy usually played in unsuccessful teams. Coaches and sports psychologists are encouraged to promote both social and task cohesion and collective efficacy to enhance team performance.

## Introduction

Several social psychological studies have shown the importance of team cohesion and the perception of efficacy as important factors in team sports (Heuzé et al., 2006; Myers et al., 2007; Paskevich et al., 1999). Moreover, some research has revealed that both variables are positively associated with performance (Carron et al., 2002; Feltz et al., 2008; Leo et al., 2010a). However, to our knowledge, no work has examined cohesion and the perception of efficacy profiles in athletes and their relationship with performance.

The Multidimensional Cohesion Model by Carron (Carron and Eys, 2012) indicated the bidirectional relationship between cohesion and collective efficacy and how this relationship can influence individual and collective team aspects. Some of these consequences are noted in this research, such as game playing (Bray and Whaley, 2001; Heuzé et al., 2006), expectations of success (Leo et al., 2010a), or performance (Heuzé et al., 2006; Ramzaninezhad et al., 2009). Hence, it would be interesting to use cluster analysis to determine different cohesion and efficacy patterns in a specific sample. This analysis could provide coaches and sports psychologists with information about the characteristics of their sports teams and thus, assist them in identifying adaptive patterns in each player.

Cohesion is understood as a “dynamic process that is reflected in part by the tendency of a group to stick together and remain united in the pursuit of its instrumental objectives and/or for the satisfaction of member affective needs” (Carron et al., 1998). The conceptual model of Carron et al. (1998) consists of four dimensions: Group integration-Task (GI-T), Group integration-Social (GI-S), Individual attraction to the group-Task (ATG-T), and Individual attraction to the group-Social (ATG-S). To create profiles according to this construct, this study divides cohesion into task and social dimensions because these dimensions have been shown to have more differences with respect to performance (Leo et al., 2010a). Carron et al.’s (2002) meta-analysis demonstrated the importance of determining whether social or task aspects were related to performance. Their work identified studies that used only two dimensions and hence demonstrated problems with the presentation of the four factors of cohesion (Heuzé et al., 2006; Leo et al., 2012). Thus, in this study, we differentiate between *task cohesion*, which reflects the degree to which group members work together to achieve common goals, and *social cohesion*, which reflects the degree to which team members empathise with each other and enjoy the group fellowship (Carron et al., 1998; Carron and Eys, 2012). These two dimensions are generated by environmental, personal, leadership and team factors that affect the perception of cohesion and produce individual and collective results, such as an influence on performance (Carron and Eys, 2012; Heuzé et al., 2006; Leo et al., 2010; Paskevich et al., 1999).

Many studies have assessed players’ and coaches’ opinions of team members’ efficacy (Bandura, 1997; Chase et al., 1997; Lent and López, 2002). Three main types of sports-related team efficacy (Beauchamp, 2007) are noteworthy: *perceived coach* e*fficacy* reflects a trainer’s confidence in a player’s abilities to perform given tasks (Beauchamp, 2007; Chase et al., 1997); *perceived peer efficacy* in sports represents players’ beliefs in their teammates’ abilities to accomplish a task successfully (Lent and López, 2002); and *collective efficacy* is a group’s shared belief in its joint ability to organise and execute the courses of action required to produce certain achievement levels (Bandura, 1997). Players form a perception of efficacy through these aspects, which lead to knowledge, affective and behavioural consequences, such as increasing or decreasing sport performance (Beauchamp, 2007; Watson et al., 2001).

Numerous investigations have found a positive relationship between both psychological constructs—cohesion and perceived efficacy—and sport performance (Heuzé et al., 2006; Kozub and McDonnell, 2000; Leo et al., 2010a; Paskevich et al., 1999; Ramzaninezhad et al., 2009; Spink, 1990; Myers et al., 2007). As previously indicated in Carron′s conceptual model, one of the consequences of achieving greater cohesion is better collective efficacy and higher performance (Carron and Eys, 2012). Most studies have found that players who perceive greater cohesion levels on their teams also perceive higher collective efficacy (Heuzé et al., 2006; Kozub and McDonnell, 2000; Leo et al., 2010a; Paskevich et al., 1999; Spink, 1990). Moreover, studies support reciprocal relationships between cohesion or collective efficacy and performance (Carron et al., 2002; Leo et al., 2012; Myers et al., 2007). Beauchamp’s (2007) collective efficacy model suggests that team cohesion is an antecedent and that performance is one of the most important consequences. Thus, most relevant studies regarding these topics have found a positive relationship with significantly high values between collective efficacy and performance (Myers et al., 2007; Watson et al., 2001). However, to our knowledge, no studies have attempted to determine the profile or degree of the cohesion and efficacy of athletes with the longest playing times (Bray and Whaley, 2001; Heuzé et al., 2006), players in teams with a higher classification (Leo et al., 2010a; Ramzaninezhad et al., 2009), or players with better performance (Heuzé et al., 2006).

Taking this aspect into account, it is interesting to examine whether players have different types of profiles regarding cohesion and perceived efficacy and how these variables influence various consequences related to team functioning. This analysis might provide important information about the most appropriate profile to achieve greater performance in a team sport. Therefore, the aim of this study is to determine the cohesion and perceived efficacy profiles of different players and to measure their differences in terms of expectations of success, playing time, and performance. As a second goal, we aimed to determine the distribution of players’ profiles in diverse teams as a function of their performance.

## Material and Methods

### Participants

The sample comprised 235 male soccer players ranging in age from 16 to 19 years old (*M* = 16.96, *SD* = .76) who were recruited from 15 affiliate teams that played in the National League in the under-18 category. Additionally, 15 coaches of the teams, whose ages ranged from 29 to 45 years (*M* = 39.93, *SD* = 4.71) and who had at least seven years of training experience in different teaching categories (*M* = 9.56, *SD* = 2.55), were selected.

All teams were recruited from the soccer league. From an original sample of 241 questionnaires collected, six (2.48%) were deleted due to invalid completion.

### Measures

#### Cohesion

An adapted Spanish version of the Group Environment Questionnaire (GEQ: Carron et al., 1985) was used to assess team cohesion. This inventory has 18 items and measures four aspects of cohesion. In this study, we were only interested in two dimensions (task and social) in an attempt to simplify the profiles into dimensions associated with performance, based on previous studies (Carron et al., 2002). Thus, task cohesion (i.e., “Our team is united in trying to reach its performance goals in training sessions and games”) and social cohesion (i.e., “Our team would like to spend time together in the offseason”) were measured. Responses were rated on a five-point Likert scale ranging from 1 (*strongly disagree*) to 5 (*strongly agree*). This study examined internal consistency through Cronbach’s alpha, indicating values of .76 for task cohesion and .73 for social cohesion.

#### Efficacy

To assess collective efficacy, peers’ perception of efficacy and coaches’ perception of efficacy, a questionnaire developed by Leo et al. (2010b) was used. We distinguished (a) *collective efficacy*, in which the athletes measured their team’s capacity; (b) *peers’ perceptions of efficacy*, in which the players assessed each other; and (c) *coaches’ perceptions of efficacy*, in which the coaches assessed their players. Responses were rated on a five-point Likert scale ranging from 1 (*strongly disagree*) to 5 (*strongly agree*). The dimensions assessed included offensive and defensive technical skills, tactical strategies, psychological aspects, and a final item of general assessment of the player (i.e., “How favourably do you evaluate this player’s defensive skills?”). All items were combined into one main factor that represented overall beliefs about the player’s efficacy in all phases of the game. This factorial structure was tested in previous works (Leo et al., 2012; Leo et al., 2010b). The scale showed alpha values of .73 for collective efficacy, .85 for self-efficacy, .80 for perceived efficacy by teammates, and .86 for perceived efficacy by coaches.

#### Success expectations

Two items were created to assess players’ beliefs in the final position that they expected to occupy and the position they thought they should occupy at the end of the season. In both cases, players chose a classification number ranging from 1 to 16. The scores were reversed so that the top rankings in the classification table (i.e., 1, 2, …) corresponded to higher scores (16, 15, …).

#### Playing time

To measure playing time, we asked how much time the athletes played in the matches. Answers were rated on a five-point Likert scale ranging from 1 (*just a little*) to 5 (*too much*).

#### Performance

To measure each team’s final performance, the final position in the classification table at the end of the regular season was used. This method of measuring performance had been employed in prior studies (Carron et al., 2002; Leo et al., 2010a; Ramzaninezhad et al., 2009). As with success expectations, we reversed the data so that better classification values (1, 2, 3, …) corresponded to higher scores (16, 15, 14, …).

### Design and Procedure

In this work, a correlation methodology with a transversal design was used. We conducted one assessment at the beginning of the season. The study received ethical approval from the University of Extremadura. All participants were treated according to the American Psychological Association’s ethics guidelines regarding consent, confidentiality, and anonymity of responses. Before the data collection, we received informed consent from the coaches, players, and players’ parents, and the general purpose of the study was explained to the participants. Data collection took place at the clubs in group settings under the supervision of trained research assistants. Participants completed the questionnaires in the changing room, for which they needed approximately 15–20 minutes. Participants completed the questionnaires individually, in the absence of their coach, supervised by the research assistants, and under non-distracting conditions.

### Data Analysis

The statistical program SPSS 19.0 was used to analyse the data and to establish sequential stages to examine the relationships between the different variables. The statistical techniques employed were factor analysis, reliability analysis, descriptive analysis, cluster analysis, analyses of variance, and analysis of contingency tables.

## Results

### Descriptive Statistics

Table 1 summarises the descriptive analysis of all the variables examined in the study. Skewness and kurtosis values were computed and revealed that the variables were reasonably normally distributed. Regarding cohesion, the means of the cohesion factors were high, although social cohesion was slightly higher than task cohesion. Moreover, considering the diverse efficacy means employed in the study, the means of collective efficacy, and peers’ perceptions of efficacy were higher than the mean coaches’ perception of efficacy, although the differences were not significant. With regard to success expectations, the participants’ mean was approximately 14 points, which suggests high success expectations in most cases.

### Cluster Analysis to Establish Cohesion and Efficacy Profiles

To determine the diverse cohesion and perceived efficacy profiles of our sample, we performed hierarchical cluster analysis including the two cohesion factors (social and task) and three efficacy factors (collective efficacy and peers’ and coaches’ perceived efficacy) in the process. The resulting dendrogram yielded four clusters as the best solution.

**Figure 1:**
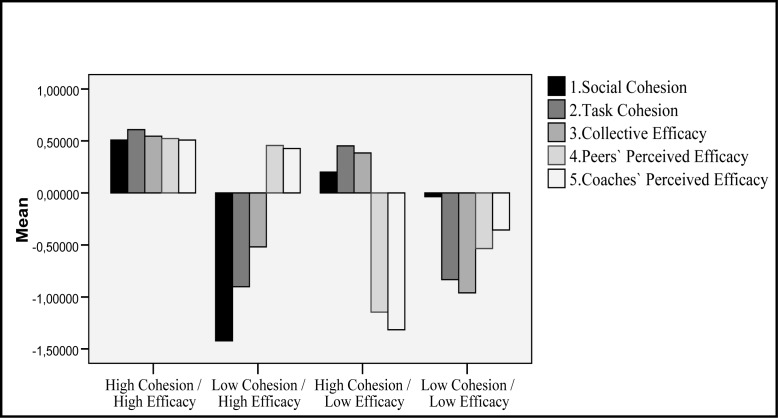
Cohesion and perception of efficacy profiles through cluster analysis.

Figure 1 shows the four profiles. The first one, High Cohesion/High Efficacy, comprised 103 participants. The second cluster comprised 41 players and corresponded with a Low Cohesion/High Efficacy profile. The third cluster comprised 39 individuals who presented a High Cohesion/Low Efficacy profile. Lastly, the fourth cluster included 52 participants with a Low Cohesion/Low Efficacy Profile.

## Analysis of Differences of Cohesion and Efficacy Profiles

Table 2 shows the differences in the diverse variables from the four profiles. A post-hoc analysis was performed to obtain more detailed information about the differences. With regard to success expectations, participants with high expectations about their final position at the end of the season were athletes with a High Cohesion/High Efficacy profile and showed significant differences from the Low Cohesion/Low Efficacy profile.

With regard to playing time differences, players with High Cohesion/High Efficacy and Low Cohesion/High Efficacy profiles played for a longer time in the matches than the Low Cohesion/Low Efficacy and High Cohesion/Low Efficacy players. Moreover, the athletes from the latter two groups thought that they should have played longer in comparison with participants from the first two groups.

Players with a High Cohesion/High Efficacy profile showed significant differences in performance compared to Low Cohesion /Low Efficacy and Low Cohesion/High Efficacy profiles. In other words, subjects whose teams finished at the highest classification had a greater perception of cohesion and were considered more efficacious by their peers and coaches, whereas players whose teams finished at the lowest classification had a low perception of cohesion regardless of their peers’ and coaches’ perceptions of efficacy.

## Contingency Table

To examine the differences in teams with high, medium, and low performance, three groups were created as a function of their position in the classification table at the end of the season. Thus, we considered the first five teams in the classification as high performance, the five middle teams as average performance, and the last five teams as the low performance group.

Furthermore, a contingency table was created by crossing the four categories of cohesion and efficacy profiles with the three group performance categories. The Pearson’s chi-square test was used to measure significance. We expected to find significant differences in the data distribution as a function of these categories. Table 3 presents the expected and observed frequencies and corrected standardised residuals. Corrected residuals over 1.9 or under −1.9 indicate that there were significantly more or less players than expected in the different performance groups. The results in Table 3 show that there were fewer low- and average-performing players than expected compared to high-performing players in the High Cohesion/High Efficacy profile. That is, there were more athletes with a High Cohesion/High Efficacy profile in high-performing teams than in low-performing teams. However, in the Low Cohesion/Low Efficacy profile, there were fewer high-performing players and more average-performing players than expected.

The value of the Pearson chi-square coefficient obtained by crossing the cohesion and efficacy profiles with the performance groups was *χ*^2^(6, *N* =235) = 13.05, *p* = .04, revealing significant differences between high-performing players in comparison with players with low and average performance.

## Discussion

The main aims of the study were to determine diverse player profiles with regard to cohesion and perceptions of efficacy and to measure the differences in them taking into consideration expectations of success, playing time, and performance. A second goal was to assess athletes’ profile distributions in each team as a function of their performance.

First, through cluster analysis, four cohesion and efficacy profiles were created: High Cohesion/High Efficacy, Low Cohesion/High Efficacy, High Cohesion/Low Efficacy, and Low Cohesion/Low Efficacy. Despite the distinction between the cohesion and efficacy profiles, cohesion and collective efficacy are grouped together in the profiles: cohesion and collective efficacy are the players’ perceptions of their own team, whereas peers and coaches are responsible for the perception of efficacy. Thus, we established different profiles for players’ perceptions and perceptions of efficacy by peers and coaches.

The differences between several profiles with regard to expectations of success, playing time, and performance were examined. We found that players who had greater success expectations for their teams were the players with a High Cohesion/High Efficacy profile, revealing significant differences from the Low Cohesion/Low Efficacy and Low Cohesion/High Efficacy profiles. Similar results were found by Chang and Bordia (2001) and Leo et al. (2010a), who reported the relationships between group cohesion, group performance, and success expectations in youth athletes. Thus, participants with higher perceptions of task cohesion showed greater confidence in group effectiveness and had higher success expectations for the group.

Regarding playing time, players with a higher perception of collective efficacy, peers’ perceptions of efficacy, and coaches’ perceptions of efficacy, regardless of cohesion, were the players with the greatest participation in the matches compared to athletes with lower efficacy levels, who thought they should play longer. These results are similar to those of Heuzé et al. (2006) who postulated that athletes with high playing time achieved better individual results (i.e., individual statistics)—that is, they were considered more efficacious and felt more involved in achieving high group cohesion to contribute to better team functioning and performance. This idea was supported by Bray and Whaley (2001) who stated that athletes with more playing time were more involved in the competition and had greater team cohesion.

Lastly, players from teams in the top final classification level were notable due to their higher perceptions of cohesion and collective efficacy, and they were perceived as more efficacious by peers and coaches. Similar outcomes were found by Ramzaninezhad et al. (2009) and Leo et al. (2013) who established that teams with better performance showed higher cohesion and collective efficacy levels. Likewise, some authors confirmed the relationship between cohesion or collective efficacy and performance (Carron and Eys, 2012; Carron et al., 2002; Leo et al., 2010a; Leo et al., 2012; Myers et al, 2007; Watson et al., 2001). In contrast, we found that players from unsuccessful teams scored lower in cohesion and collective efficacy than athletes from successful teams, regardless of the perception of efficacy by peers and coaches. Leo et al. (2012) also found that low-performing teams had a lower perception of cohesion and collective efficacy (Ramzaninezhad et al., 2009).

Our second aim was to measure the player’s profile distribution in different teams as a function of performance. There were more athletes in high-performing teams with High Cohesion/High Efficacy profiles; that is, there were more players who perceived greater cohesion and collective efficacy and who were, in turn, perceived by their peers and coaches as more efficacious in high-performing teams than in low- or average-performing teams. In this sense, as mentioned, Leo et al. (2013) also found that players in successful teams scored higher in perceptions of cohesion and collective efficacy. There were fewer athletes with a Low Cohesion/Low Efficacy profile in successful teams and more in average teams. In other words, there were fewer players with lower cohesion and collective efficacy and lower perceived peer and coach efficacy in high-performing teams than in average- and low-performing teams. These results are consistent with those of Ramzaninezhad et al. (2009) who indicated that players in unsuccessful teams tended to rate their teams with lower cohesion and collective efficacy levels (Leo et al., 2013). Moreover, many researchers have claimed that players who perceive greater cohesion levels in their teams also perceive higher collective efficacy (Kozub and McDonnell, 2000; Leo et al., 2010a; Paskevich et al., 1999; Spink, 1990) and achieve better performance (Leo et al., 2012).

## Conclusions, Future Prospects, and Limitations

One of the most important conclusions of this study is that team sports include players with different profiles in terms of perceptions of cohesion and efficacy. In this regard, athletes with higher cohesion and efficacy profiles showed better expectations, played more time in games, and belonged to teams with better performance. Another conclusion of this study is that teams at the beginning of the season that had more players with high cohesion and efficacy profiles finished at the top classification; in contrast, athletes with low cohesion and collective efficacy profiles usually played in unsuccessful teams.

Hence, the principal practical implication of this study is that coaches may attempt to identify players with high cohesion and collective efficacy profiles at the beginning of the season to help the team achieve better performance. Thus, we propose that coaches and sports psychologists should promote either social and task cohesion and collective efficacy. With respect to group cohesion, some of the most frequent strategies are based on social aspects, such as scheduling meetings, organising multisport activities, or going out for dinner. However, few strategies have been used in training to improve task cohesion, such as establishing group goals (Senécal et al., 2008), planning responsibilities and communication tasks during training sessions (Leo et al., 2009), or providing guidelines during training sessions to enhance the team (Spink and Carron, 1993).

Other noteworthy strategies to maintain or increase collective efficacy levels throughout a season might include the development of an adequate motivational climate, clarification of team roles, identifying the coaches’ and most important players` leadership capacity, or maintaining appropriate team expectations (Beauchamp, 2007).

Lastly, prospects for the future include the development of the above-mentioned strategies in intervention programmes to improve cohesion and collective efficacy and to achieve better performance in team sports. As a limitation of the study, we note that the participants were all youths. Although they were performance-oriented, they were still at the learning stage, which could influence the relationships between the variables and performance.

## Figures and Tables

**Table 1:** Descriptive statistics

	*M*	*SD*	*MIN*	*MAX*	*SK*	*CK*	*α*
Social Cohesion	4.06	.70	1.20	5.00	−.92	1.28	.73
Task Cohesion	3.75	.71	1.50	5.00	−.58	.29	.76
Collective Efficacy	3.80	.54	1.86	5.00	−.29	−.10	.73
Peers’ Perception of Efficacy	3.73	.39	2.44	5.00	−.67	.74	.80
Coaches’ Perception of Efficacy	3.51	.71	1.00	5.00	−.11	.19	.86
Expectations of success	14.50	2.36	6	16	−1.69	1.20	-
Playing Time	3.97	1.11	1	5	−1.08	.56	-

**Table 2:** Analysis of variance through cohesion and efficacy profiles

	High Cohesion/High Efficacy	Low Cohesion/High Efficacy	High Cohesion/Low Efficacy	Low Cohesion/Low Efficacy	*MC*	*F*	*p*
Expectations	15.10±1.79	14.24±2.60	13.96±2.82	13.94±2.56	22.48	4.21	.01
Playing Time	4.42±.73	4.12±1.10	3.13±1.34	3.60±1.11	18.82	18.56	.00
Performance	9.98±4.48	7.76±4.00	8.59±4.38	8.02±3.86	72.61	4.02	.01

**Table 3:** Contingency table of cohesion and efficacy profile by performance

		Performance			
		High	Medium	Low	Total
High Cohesion/High Efficacy	*N*	43	31	29	103
	Expected Frequency	32.4	35.9	34.6	103
	Corrected Residual	3.0	−1.4	−1.6	
Low Cohesion/High Efficacy	*N*	8	16	17	41
	Expected Frequency	12.9	14.3	13.8	41
	Corrected Residual	−1.8	.6	1.2	
High Cohesion/Low Efficacy	*N*	13	11	15	39
	Expected Frequency	12.3	13.6	13.1	39
	Corrected Residual	.3	−1.0	.7	
Low Cohesion/Low Efficacy	*N*	10	24	18	52
	Expected Frequency	16.4	18.1	17.5	52
	Corrected Residual	−2.2	1.9	.2	
